# Factors Influencing Recruitment, Retention, and Adherence Rates in Exercise Interventions in ESKD: A Scoping Review

**DOI:** 10.1016/j.xkme.2024.100933

**Published:** 2024-11-14

**Authors:** Malvika Agarwal, Jamie Alexiuk, Clara Bohm, Lindsey Sikora, Deborah Zimmerman

**Affiliations:** 1 Faculty of Medicine, University of Ottawa, Ontario, Canada; 2 Faculty of Kinesiology, University of Manitoba, Manitoba, Canada; 3 Section of Nephrology, Department of Internal Medicine, University of Manitoba, Manitoba, Canada; 4 Kidney Research Centre, The Ottawa Hospital, Ontario, Canada; 5 Department of Nephrology, The Ottawa Hospital, Ontario, Canada

**Keywords:** Adherence, dialysis, end-stage kidney disease (ESKD), exercise, recruitment, retention

## Abstract

**Rational & Objective:**

Majority of people with end-stage kidney disease (ESKD) are sedentary, which increases risk for decreased quality and quantity of life. Development of exercise programs with characteristics that address individual preferences may increase interest in participating and completing exercise programs. We evaluated which exercise intervention characteristics affect exercise program recruitment, adherence, and completion in people with ESKD receiving dialysis.

**Study Design:**

Scoping review of randomized controlled trials (RCTs) with searching of Medline, Embase, Cochrane, and CINAHL databases through May 12, 2023.

**Setting & Study Populations:**

Adults with ESKD receiving dialysis.

**Selection Criteria:**

RCTs with exercise interventions of ≥12 weeks that included more than 10 people with ESKD receiving dialysis in each study arm.

**Data Extraction:**

One individual extracted data and the second author checked for accuracy.

**Analytical Approach:**

Data were synthesized qualitatively. Associations between intervention characteristics and recruitment, retention and adherence rates were assessed through one-way analysis of variance tests. Risk of bias was assessed using the Cochrane Risk of Bias 1.0 tool.

**Results:**

Of 7,396 studies identified, 55 studies with 3,269 trial participants were included. The majority of participants were male (63.2%) and treated with hemodialysis (89.1%). Mean age was 56 ± 11.5 years. Average recruitment, retention and adherence rates were 77.4%, 81.2%, and 76.0%, respectively. Only 27% of studies reported adherence rates. No significant associations were found between intervention characteristics (ie, exercise type, duration, frequency, setting, and supervision) and recruitment, adherence, and retention rates.

**Limitations:**

Data were limited by small sample size, suboptimal risk of bias, selective recruiting methods, and variability in definitions of adherence rates.

**Conclusion:**

Average recruitment, retention, and adherence rates in exercise interventions for patients receiving dialysis were high although less than 1 in 4 studies reported adherence rates. These results call for standardized reporting of recruitment, retention, and adherence rates in exercise interventions.

## Plain-Language Summary

Regular exercise for people with end-stage kidney disease (ESKD) can improve their quality of life. We sought to determine if there were certain characteristics of an exercise program such as exercise type, frequency, program length, and setting that were associated with enhanced recruitment, retention, and adherence to the program. From the 55 identified studies, average recruitment, retention, and adherence rates in exercise interventions were high irrespective of the program characteristics. However, it is unclear if our results are robust as there was a lack of formalized definitions for recruitment, retention, and adherence rates. Moreover, very few studies reported adherence rates. Therefore, we suggest that standardized definitions and reporting of these factors be required in exercise studies.

End-stage kidney disease (ESKD) is associated with high mortality; globally there were 3.2 million deaths and 76.5 million disability-adjusted life years attributable to the disease in 2019.^1^ Several factors are responsible for the decreased survival and quality of life in people with ESKD receiving dialysis including high burden of comorbid disease, dialysis-related side effects, and overwhelming fatigue, all of which contribute to markedly impaired physical performance and functioning.^2^ Low physical activity is associated with adverse outcomes and poorer mental health.^3^ On the contrary, people who exercise regularly have higher quality of life scores, improved muscle strength, improved cardiovascular function, and reduced hospitalization rates and symptoms of anxiety and depression.^4^^,^^5^ There is also growing interest in the role of regular exercise as a strategy for "prehabilitation" of patients with ESKD before kidney transplantation.^6^ Because of the reported benefits of exercise, the National Kidney Foundation Kidney Disease Outcomes Quality Initiative (NKF KDOQI) clinical practice guidelines state that people with ESKD “should be counselled and regularly encouraged….to increase their level of physical activity” (grade B) but it is unclear how to best achieve clinically important increases in physical activity in this population.^7^

Despite the potential benefits, most people with ESKD are sedentary and do not participate in regular physical activity. In a study using data from the Dialysis Outcomes and Practice Patterns Study (DOPPS), 43.9% (n = 9,176) of people treated with hemodialysis did not engage in regular exercise.^8^ For some individuals, nonmodifiable barriers to exercise may exist such as a high burden of comorbid illnesses that may make it unsafe or difficult to participate in formal exercise programs. However, for others, modifiable barriers such as knowledge about the benefits of exercise, or development of exercise programs with characteristics that address individual preferences may increase interest in participating and completing such programs.^9^ These exercise program characteristics may include but are not limited to intervention length, exercise frequency, exercise type, setting, supervision, and incorporation of motivational strategies.

To date, it is unknown what exercise intervention characteristics influence recruitment, retention and intervention adherences rates in exercise trials for people with ESKD. Such knowledge is critical to assist with design and implementation of exercise interventions for clinical trials and the development of programs for incorporation into clinical kidney care. To address this knowledge gap, we examined exercise intervention characteristics and their impact on exercise program participation, completion, and adherence in people with ESKD receiving dialysis.

## Methods

This scoping review was performed in accordance with Preferred Reporting Items for Systematic Reviews and Meta-analyses guidelines and the protocol was registered in Open Science Framework (DOI:10.17605/OSF.IO/HVU8E). The following databases were searched on May 12, 2023, by a health sciences librarian (LS): MEDLINE and MEDLINE in Process via Ovid, Embase Classic + Embase via Ovid, Cochrane CENTRAL via Ovid, and CINAHL via EBSCOhost. A search strategy was developed in MEDLINE and then translated into the other databases as appropriate. A randomized controlled studies filter was used in the Medline, CINAHL, and Embase databases (CADTH, 2023). The Medline search strategy was peer reviewed using the Peer Review of Electronic Search Strategies (PRESS) tool by Evan Sterling (Research Librarian, Health Sciences and Medicine). All databases were searched from dates of inception to May 12, 2023. All references were entered into an Endnote file for processing and then uploaded into Covidence for duplicate removal and screening. References from 2 recent systematic reviews/meta-analysis were also searched.^10^^,^^11^

Two individual authors (MA and JA) screened titles and abstracts for eligible articles. The inclusion criteria included: (a) randomized controlled trials (RCTs); (b) adults (>18 years of age) with ESKD treated with dialysis; (c) exercise interventions of ≥12 weeks with at least 10 participants in each arm; (d) control group received usual/standard care (no additional intervention) and reported at least one of the following prespecified outcomes: recruitment rate, retention rate, and adherence rate. Recruitment was calculated variably based on the information provided by authors in which all eligible patients in the dialysis unit versus only patients approached was used as the denominator; the number of consenting patients was used as the numerator. Retention was defined as the number of individuals who participated in the exercise intervention to completion as a proportion of the total number of participants recruited. Adherence rates were also variably calculated based on author definition, including proportion of participants who attended sessions, proportion of participants compliant with the entire prescribed exercise, or proportion of participants who achieved the target workload of the program.

If the study flow diagram or description did not include the number of individuals screened and/or those who declined participation, the study was not included in the calculation of recruitment rate. Studies were excluded if they met the following criteria: (a) there were multiple comparison groups (ie, more than one control or intervention group); (b) cointervention in the control group that was not consistent with a “placebo” (ie, nutritional supplementation use); (c) abstract only; and (d) non-English language. If there were multiple studies reporting the same patient population, only one study was included. We excluded studies with multiple comparison groups because majority of exercise trials cannot be blinded, therefore having multiple groups might influence participant behaviors if they notice others are performing a different intervention than them. Full-text screening was conducted by MA and JA. Any discrepancies for eligibility in the title/abstract and/or full-text screening stages were discussed by authors until a final consensus was reached with input from the other authors (CB, DZ).

One author (MA or JA) extracted data from the included studies, and the second author verified the extracted data. Any disagreements were resolved through consensus (MA, JA, CB, DZ). Extracted information included the following: (a) study title; (b) authors; (c) year of publication; (d) number of participants overall and in each study arm; (e) eligibility criteria for study participants; (f) country; (g) aggregate participant demographics (sex, age, cause of ESKD, comorbid conditions, dialysis modality, duration of dialysis); (i) blinding method; (j) study outcomes. We collected the following outcomes for our review: recruitment rate (%), retention rate (%) and adherence rate (%). Exercise intervention characteristics were extracted using the Consensus on Exercise Reporting Template (CERT) (Table S1).^12^

Risk of bias was assessed using the Cochrane Risk of Bias tool 1.0 for RCTs. Disagreements were resolved through consensus between reviewers. Bias was assessed in 5 distinct domains: bias arising from the randomization process, bias because of deviations from intended interventions, bias because of missing outcome data, bias in the measurement of the outcome (blinding) and bias in the selection of the reported result. Within each domain, the 2 reviewers answered one or more signaling questions (eg, Was the allocation sequence random? Were people aware of their assigned intervention during the trial?) that led to judgments of “low risk of bias,” “some concerns,” or “high risk of bias.” The judgments within each domain led to an overall risk-of-bias judgment for the result being assessed.

Review findings were synthesized qualitatively through description of exercise interventions that report recruitment, retention and adherence rates. One-way analysis of variance (ANOVA) was performed to assess association of recruitment, retention and adherence rates with exercise intervention characteristics. We reported means, standard deviations, and associated *P* values with 95% confidence intervals.

## Results

**Figure 1:**
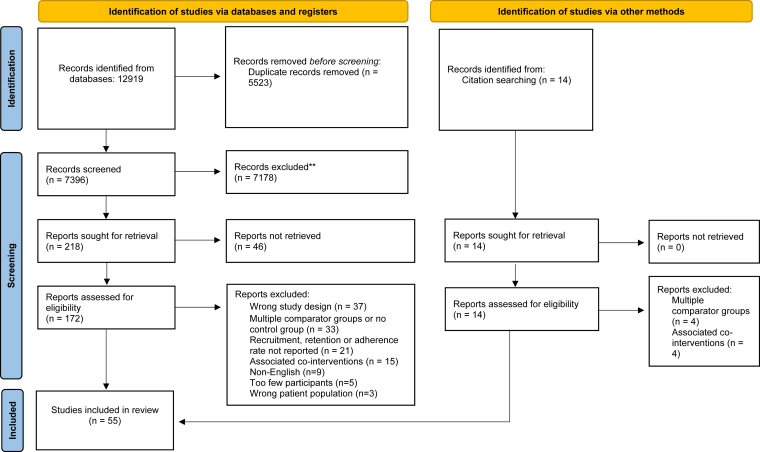
Preferred Reporting Items for Systematic Reviews and Meta-analyses flowsheet of publications identified, included and excluded, and the reasons for exclusions.

The database search yielded 12,919 articles, of which 5,523 were excluded as duplicates (Fig 1). Title and abstract screening was performed for 7,396 articles, resulting in 218 remaining papers for full-text screening. Initially, 49 randomized control trials were included in our study. Studies were excluded for the following reasons: (a) abstract only (n = 46), (b) non-English (n = 9), (c) multiple comparator groups or no control group (n = 33), (d) associated co-interventions (n = 15), (e) non-RCTs (n = 37), (f) insufficient number of participants (n = 5), (g) non-ESKD patients (n = 3), and (h) no relevant outcomes reported (n = 21). After review of references from 2 recent meta-analyses, an additional 14 manuscripts were screened and 6 were retained, leading to a final inclusion of 55 randomized control trials in our study (Tables S2 and S3).^10^^,^^11^^,^^13^, ^14^, ^15^, ^16^, ^17^, ^18^, ^19^, ^20^, ^21^, ^22^, ^23^, ^24^, ^25^, ^26^, ^27^, ^28^, ^29^, ^30^, ^31^, ^32^, ^33^, ^34^, ^35^, ^36^, ^37^, ^38^, ^39^, ^40^, ^41^, ^42^, ^43^, ^44^, ^45^, ^46^, ^47^, ^48^, ^49^, ^50^, ^51^, ^52^, ^53^, ^54^, ^55^, ^56^, ^57^, ^58^, ^59^, ^60^, ^61^, ^62^, ^63^, ^64^

Included studies were conducted worldwide with 36.4% (n = 20) from Asia, 20.1% (n = 11) from South America, 25.5% from Europe (n = 14), 16.4% (n = 9) from North America, and 1.8% (n = 1) from Australia. The total number of participants from all the included studies was 3m269, 1,645 in the exercise group, and 1,624 in the control group (Table 1). The average number of participants per study was 30 per group. The average age of participants in the intervention and control group was 56.5 years (standard deviation [SD] ± 11.8) and 56.4 (SD ± 11.4), respectively. The majority of the study population was male (63.2%) and treated with hemodialysis (89.1%). Of the studies reporting comorbid conditions, diabetes mellitus was present in 32.6% versus 36.4%, hypertension in 57.3% versus 53.9%, and cardiovascular disease 25.9% versus 24.2% in the intervention and control groups, respectively (Table 2).

**Table 1:** Exercise Intervention Characteristics

Intervention Characteristics	% (n)
Mean duration (wk)	20
Median duration (wk)	16
**Exercise type (%)**	
Aerobic	38.2 (n = 21)
Resistance	21.8 (n = 12)
Combined aerobic/resistance	34.5 (n = 19)
Other (ie, yoga, Bandujan)	5.5 (n = 3)
**Duration (%)**	
≤12 wk	49.1 (n = 27)
13-23 wk	12.7 (n = 7)
≥24 wk	38.2 (n = 21)
**Supervision (%)**	
Supervised	70.9 (n = 39)
Nonsupervised	16.4 (n = 9)
Unspecified	12.7 (n = 7)
**Setting (%)**	
Hospital	70.9 (n = 39)
Home	10.9 (n = 6)
Other (ie, gym, rehab room, clinic)	7.3 (n = 4)
Unspecified	10.9 (n = 6)
**How was exercise performed? (%)**	
Individually	71.8 (n = 41)
Group	42.9 (n = 11)
Unspecified	5.5 (n = 3)
**Exercise frequency (%)**	
2 times/wk	12.7 (n = 7)
3 times/wk	76.4 (n = 42)
>3 times/wk	9.1 (n = 5)
Unspecified	1.8 (n = 1)
**Generic or tailored? (%)**	
Generic	80.0 (n = 44)
Tailored	16.4 (n = 9)
Not specified	3.6 (n = 2)
**Use of motivation strategies (%)**	
Yes	12.7 (n = 7)
No	87.3 (n = 48)

**Table 2:** Participant Characteristics

Column1	Intervention Group	Control Group
**Total number of participants**	1,645	1,624
**Average number of participants**	30 ± 24	30 ± 25
**Males (%)**	62.2 ± 19.0	64.2 ± 17.5
**Average age (y)**	56 ± 12	56 ± 11
**Average duration of dialysis (mo)**	56.0 ± 32.4	58.4 ± 32.3
**Dialysis modality (number of participants)**		
Hemodialysis	1531	1500
Peritoneal dialysis	114	124
**Comorbid conditions (%)**		
Hypertension	57.3 ± 29.7	53.9 ± 31.8
Diabetes	32.6 ± 15.0	36.4 ± 18.3
Heart disease	25.9 ± 15.0	24.2 ± 10.5

### Intervention Descriptions

Most studies (38.2%; n = 21) employed aerobic exercises. In total, 34.5% (n = 19) were combined aerobic and resistance, and 21.8% (n = 12) were resistance-based exercises (Table 1). Other exercise interventions included yoga and Bandujan (combined breathing, body movement and meditation).^65^^,^^66^ In one intervention, participants self-selected activities such as household chores, gardening, or walking as part of the exercise regimen.^67^ Intervention length ranged from 12 to 104 weeks. In total, 49.1% (n = 27) of interventions were 12 weeks, 12.7% (n = 7) were between 13 and 23 weeks, and 38.2% (n = 21) were greater than 24 weeks. Most of the studies were supervised by a professional (70.9%; n = 39) and conducted in a hospital setting (70.9%; n = 39). Six studies (10.9%) were conducted in a home setting. Forty-two studies (76.4%) had an exercise program frequency of 3 times per week, 7 studies (12.7%) were held 2 times per week, 5 studies (9.09%) were more than 3 times a week, and the remaining 1 study (1.82%) did not specify frequency. Exercise duration ranged from 30 to 120 minutes per session. Almost all the studies (80.0%; n = 44) had generic, predesigned exercise interventions that were not modified to patient’s personal preferences. Exercise intensity varied based on type of exercise and the type of intensity scale used. Seven studies (12.7%) used motivational strategies.

### Recruitment, Retention, and Adherence Rates and Associations

Among the included studies, 76% (n = 42) reported recruitment rates, 98% (n = 54) reported retention rates, and 27% (n = 15) reported adherence rates (Table 3). The average recruitment, retention and adherence rates among the included studies reporting these outcomes was 67.7%, 80.7%, and 75.1% respectively.

**Table 3:** Recruitment, Retention, and Adherence Rates

	Recruitment	Retention	Adherence
Mean	67.7	80.7	75.1
Standard deviation	22.8	12.6	19.7
% of Studies reported (n)	76% (n = 42)	98% (n = 54)	27% (n = 15)

No significant associations were found between intervention characteristics (ie, exercise type, intervention length, intervention setting, frequency, supervision, intervention duration and whether exercises are in groups) and recruitment (Table 4), retention (Table 5), and adherence (Table 6) rates. All *P* values were greater than 0.05.

**Table 4:** Recruitment Rate Association With Different Intervention Characteristics^a^

Variable	Average Recruitment Rate (%)	N	Standard Deviation	*P* Value^b^
**Exercise type**				
Aerobic	62.7	14	23.7	0.58
Resistance	65.6	10	21.0	
Combined Aerobic/Resistance	73.0	16	23.3	
Other	63.1	2	24.7	
**Intervention length (wk)**				
12	66.7	20	20.8	0.96
13-23	69.2	6	26.5	
24 or more	68.5	16	25.4	
**Intervention setting**				
Hospital	68.4	31	24.1	0.80
Home	70.8	6	17.6	
Other	58.2	2	13.2	
**Frequency (times/wk)**				
Less than 3	69.6	30	20.7	0.90
3	69.2	5	24.5	
More than 3	74.1	5	20.9	
**Supervision**				
Supervised	68.4	28	24.3	0.57
Nonsupervised	63.2	9	21.9	
**How exercises are performed**				
Individually	67.1	31	23.3	0.16
Group	75.9	8	15.0	
**Intervention duration (min)**				
30 min or less	70.9	10	21.6	0.76
31-59 min	65.7	14	24.4	
60 min or greater	71.7	15	21.8	

Note: Results of one-way ANOVA test comparing means of predictors (ie, exercise type, intervention length, intervention setting, frequency, supervision, how exercises are performed, intervention duration) with recruitment rates.

a Some studies did not provide information on certain intervention characteristics and were thus excluded from the ANOVA analysis (ie, 3 studies did not state the setting of their intervention therefore, only 39 studies were included in the ANOVA analysis).

b 95% confidence interval (CI).

**Table 5:** Retention Rate Association With Different Intervention Characteristics^a^

Variable	Average Retention Rate	N	Standard Deviation	*P* Value^b^
**Exercise type**				
Aerobic	79.4	22	14.1	0.41
Resistance	77.0	11	15.1	
Combined aerobic/resistance	84.5	19	8.8	
Other	83.1	3	11.8	
**Intervention length (wk)**				
12	80.9	27	12.9	0.67
13-23	76.7	7	20.2	
24 or more	81.7	20	9.1	
**Intervention setting**				
Hospital	83.7	38	9.9	0.15
Home	76.6	6	8.9	
Other	77.5	4	7.4	
**Frequency (times/wk)**				
Less than 3	81.1	41	11.7	0.51
3	79.9	6	22.4	
More than 3	74.1	5	5.4	
**Supervision**				
Supervised	80.8	39	13.2	0.32
Nonsupervised	76.1	9	9.7	
**How exercises are performed**				
Individually	79.4	41	11.7	0.23
Group	84.8	10	16.6	
**Intervention duration (min)**				
30 min or less	82.4	14	10.6	0.89
31-59 min	81.9	16	11.8	
60 min or greater	80.6	22	12.7	

Note: Results of 1-way ANOVA test comparing means of predictors (ie, exercise type, intervention length, intervention setting, frequency, supervision, how exercises are performed, intervention duration) with recruitment rate.

a Some studies did not provide information on certain intervention characteristics and were thus excluded from the ANOVA analysis (ie, 3 studies did not state the setting of their intervention therefore, only 39 studies were included in the ANOVA analysis).

b 95% confidence interval (CI).

**Table 6:** Adherence Rate Association With Different Intervention Characteristics^a^

Variable	Average Adherence Rate	N	Standard Deviation	P Value^b^
**Exercise type**				
Aerobic	68.7	5	31.12	0.794
Resistance	83.08	4	10.97	
Combined aerobic/resistance	74.76	5	13.52	
Other	77.10	1	11.80	
**Intervention length (wk)**				
12	74.90	6	13.87	0.743
13-23	60.00	1	19.64	
24 or more	77.15	8	25.50	
**Intervention setting**				
Hospital	74.86	9	24.24	0.966
Home	72.28	4	13.80	
Other	78.00	1	20.2	
**Frequency (times/wk)**				
Less than 3	52	11	21.53	0.530
3	75.05	1	10.34	
More than 3	80.05	2	4.17	
**Supervision**				
Supervised	77.75	10	22.43	0.241
Nonsupervised	69.82	5	13.16	
**How exercises are performed**				
Individually	73.31	11	22.37	0.786
Group	77.07	3	9.53	
**Intervention duration (min)**				
30 min or less	75.33	4	16.32	0.086
31-59 min	55.85	4	26.36	
60 min or greater	85.42	5	7.44	

Note: Results of one-way ANOVA test comparing means of predictors (ie, exercise type, intervention length, intervention setting, frequency, supervision, how exercises are performed, intervention duration) with recruitment rate.

a Some studies did not provide information on certain intervention characteristics and were thus excluded from the ANOVA analysis (ie, 3 studies did not state the setting of their intervention therefore, only 39 studies were included in the ANOVA analysis).

b 95% confidence interval (CI).

### Quality Assessment

**Figure 2:**
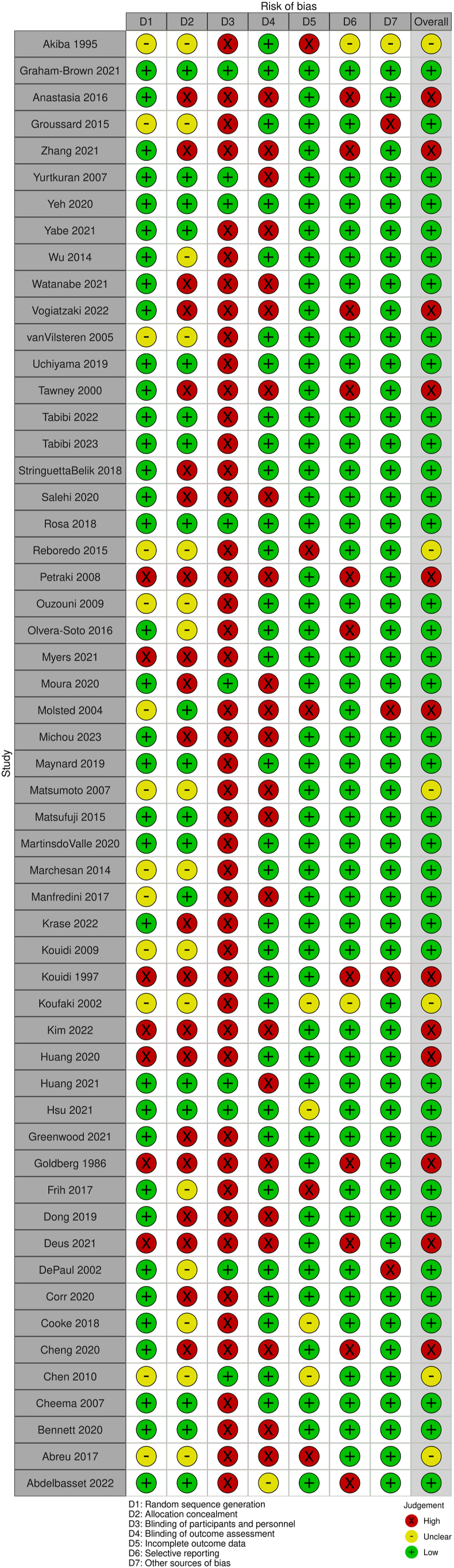
Risk of bias summary for each included study (using Cochrane Risk of Bias tool 1.0).

**Figure 3:**
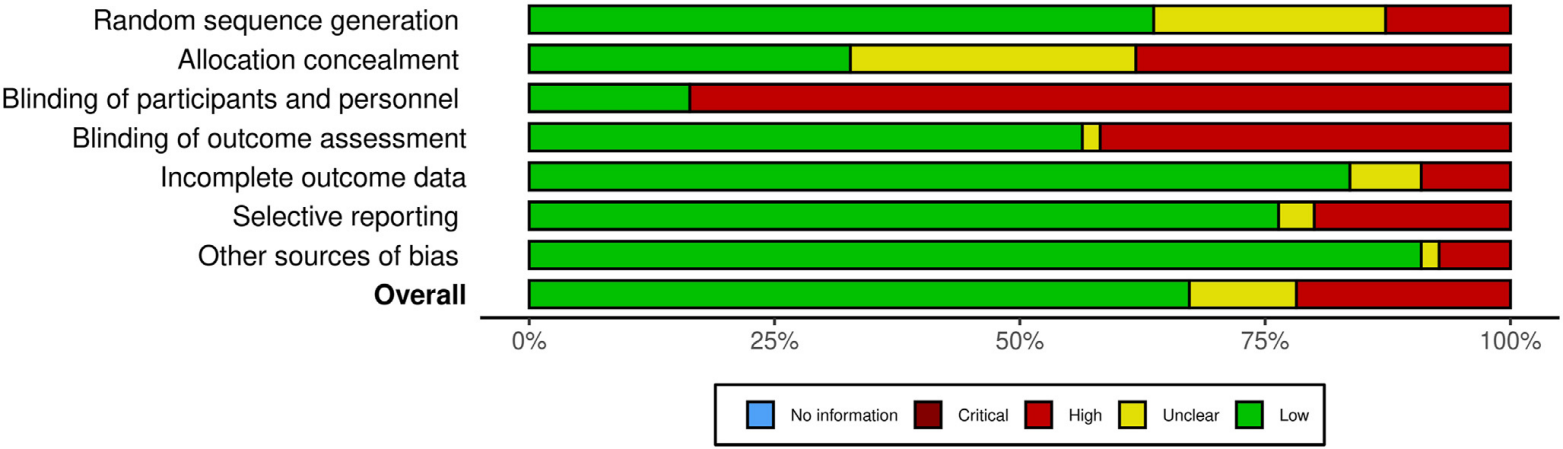
Risk of bias graph presented as percentages across all included studies (using Cochrane Risk of Bias tool 1.0).

Quality assessment of the included studies is shown in Figure 2, Figure 3. After assessment of the Cochrane ROB tool, overall risk of bias based on combination of all criteria was low for 35 studies, high for 7 studies, and unclear for 13 studies. Random sequence generation and the method of concealment was a low risk of bias for 35 and 18 studies, respectively. Complete blinding of participants was unlikely because of the nature of exercise intervention trials; however, we deemed 9 studies to have low risk of bias because of implementation of a placebo or sham exercise. Low risk of bias was present in 9 studies for blinding of outcome assessors and 4 studies for incomplete outcome data. Other potential sources of bias were deemed to be high in 8 studies because they received private funding but did not provide further information about funder involvement.

## Discussion

In our scoping review of the literature, we included 55 studies with 3,269 participants for which we were able to extract recruitment, retention, and/or adherence rates. Overall, mean recruitment, retention, and adherence rates were 67.7%, 80.7%, and 75.1%, respectively. No significant associations were found between exercise intervention characteristics and recruitment, retention, and adherence rates.

A study published in 2021 comparing exercise trial characteristics in multimorbid populations also did not find any associations with recruitment and retention rates.^68^ It is possible that well designed exercise trials are equally effective at recruitment and retention; however, it is equally possible that a lack of standardized reporting for recruitment, retention and adherence underpins this lack of association.

Recruitment rates were only reported in three quarters of the included studies. Mean overall recruitment rate for these studies was consistent with some but not all reviews of exercise trials in other chronic disease populations. Median recruitment rates in RCTs of exercise were 75% in people with multimorbidity and 38% in people living with cancer.^68^^,^^69^ Globally, 55% of clinical trials have been terminated because of low recruitment rates; however, the proportion of ESKD exercise trials that have terminated for similar reasons is unknown.^68^ In our review, it is unclear if recruitment rates in patients with ESKD are truly higher than in patients living with cancer or because of differences in calculation and reporting of recruitment rates. In many studies included in our review, the number of individuals in a center who were eligible to participate in the trial was unclear. Many of the trials that did not report recruitment rates (92% [n = 12]) were published before 2017. This lack of standardization in reporting of recruitment in earlier publications is consistent with the changes in reporting of recruitment over time required by scientific journals (eg, CONSORT statement).^70^ However, the recruited participants in our review were not representative of the general dialysis population with an average age of only 56 (SD ± 12) years and 63.2% males. Reasons for this could be that only healthier and younger individuals were approached for recruitment or that recruiting individuals that are more representative of the general ESKD population (ie, older, lower functional status) was difficult. Ultimately, this could have resulted in lower recruitment rates than those observed in other studies of multimorbid patients. When healthier individuals are recruited, the results may not be generalizable. The average number of participants per group in our scoping review was only 30, limiting the ability to detect statistically significant clinically relevant changes as sample size calculations were frequently not included in the methods.

The retention rate in our study was greater than 80%, which is in keeping with previously reported retention rates of exercise trials among people with chronic obstructive pulmonary disease (82%), heart failure (97%), depression (100%) and multimorbidity in general (90%)(16). It is important to consider that factors aside from the exercise intervention can also affect retention rates (ie, required commitment to the program, fatigue, peer pressure, reimbursement for study participation and completion). Although some prior studies have shown increased retention to home-based, moderate intensity and supervised exercise programs, this was not supported by our findings.^69^^,^^70^ Similarly, previous studies have also found no observed difference between retention rates and exercise intervention characteristics.^71^^,^^72^ The use of motivational strategies to increase participant retention in exercise programs has been effective in previous studies.^73^ However, only 16% of our included studies employed some form of behavioral change strategy, and of those that did, the majority were done in programs that were unsupervised or home based. The strategies included regular phone calls, emails to participants, follow-up during clinic visits and counseling sessions. It is important to consider that participants in supervised trials may have also received consistent encouragement which could be considered a motivational strategy. Further exploration into the effectiveness of varying motivational strategies can provide insight on how they can be incorporated in exercise programs. Lastly, multiple exercise program characteristics make it difficult to separate out the individual effects of program components such as supervised aerobic exercise in the hemodialysis unit or a home-based program with motivational strategies included for people treated with peritoneal dialysis.

For an exercise program to have the potential to improve outcomes, participants need to be adherent to the exercise intervention. The majority of included studies did not report adherence. This could be attributed to lack of a standard definition or method for accurately measuring adherence among exercise trials. For those studies that did report adherence, multiple definitions were used among different studies including attendance at sessions, completion of goal exercise duration at each session, achieved target workload or completed the requirements of the exercise program. This variability hinders accurate comparisons and collective conclusions. Poor adherence reporting fails to capture important components of an exercise program such as the percentage able to complete the exercise program. Such information is required to facilitate the design of feasible exercise programs. Furthermore, many of the studies did not provide detailed protocols or a system of decision rules for the exercise programs, which may have contributed to underreporting of detailed adherence data. It then becomes impossible to know if an exercise trial did not have beneficial outcomes because of a lack of adherence, the need for a greater volume/intensity of exercise or because of true lack of efficacy for the study endpoint.

### Strengths and Limitations

This is the first review to quantify recruitment, retention and adherence rates and associated participant and intervention characteristics in ESKD exercise trials. We conducted an extensive systematic search of the literature with the help of a librarian to identify potentially relevant studies. We included RCTs of sufficient duration and participant numbers to allow for meaningful conclusions. Selection bias was minimized by 2 author independent review of the studies.

Our study has several limitations. We included studies that were designed and published before standardized reporting, thereby limiting our ability to identify recruitment rates for some of the studies. Recruitment rates that were reported as 100% were not included in the average rate as there were concerns about patient selection strategies. The inclusion of English only studies and those studies with only 2 groups (intervention/control) may have resulted in the exclusion of relevant publications. Furthermore, the small number of studies in our review, high risk of bias in some articles and variability in the definition of adherence may have led to limited power and generalizability, therefore hindering our ability to detect different associations with intervention characteristics and our chosen outcomes.

### Implications for Future Research

It remains unclear whether certain exercise characteristics can enhance recruitment, retention and adherence rates in ESKD exercise trials. This uncertainty can be partially attributed to lack of standardized reporting methods and variability in adherence definitions. It is important that future exercise trials describe detailed and transparent definitions and methods for recruitment, retention, and adherence to facilitate improved implementation and sustainability of exercise programs in both research and clinical kidney care programs. For example, Castillo et al^74^ developed a feasibility assessment tool for implementation of intradialytic exercise interventions. Similar tools can assist with designing multisite evidence-based structured exercise programs that are more likely to have high patient interest and engagement.

## Conclusion

In this scoping review of exercise trials in patients with ESKD, we were unable to identify specific characteristics of the exercise intervention that were associated with enhanced recruitment, retention and adherence rates. However, we have identified several areas of concern that require improved standardization of reporting including the accuracy of the denominator in recruitment and the importance of consistent adherence reporting overall.

## References

[1] Zhang S., Ren H.F., Du R.X., Sun W.L., Fu M.L., Zhang X.C. (2023). Global, regional, and national burden of kidney dysfunction from 1990 to 2019: a systematic analysis from the global burden of disease study 2019. BMC Public Health.

[2] Kaysen G.A., Larive B., Painter P. (2011). Baseline physical performance, health, and functioning of participants in the Frequent Hemodialysis Network (FHN) trial. Am J Kidney Dis.

[3] Stack A.G., Molony D.A., Rives T., Tyson J., Murthy B.V.R. (2005). Association of physical activity with mortality in the US dialysis population. Am J Kidney Dis.

[4] Baker L.A., March D.S., Wilkinson T.J. (2022). Clinical practice guideline exercise and lifestyle in chronic kidney disease. BMC Nephrol.

[5] Zheng J., You L.M., Lou T.Q. (2010). Development and psychometric evaluation of the Dialysis patient-perceived Exercise Benefits and Barriers Scale. Int J Nurs Stud.

[6] McAdams-DeMarco M.A., Ying H., Van Pilsum Rasmussen S. (2019). Prehabilitation prior to kidney transplantation: Results from a pilot study. Clin Transplantat.

[7] (2005). K/DOQI clinical practice guidelines for cardiovascular disease in dialysis patients. Am J Kidney Dis.

[8] Tentori F., Elder S.J., Thumma J. (2010). Physical exercise among participants in the Dialysis Outcomes and Practice Patterns Study (DOPPS): correlates and associated outcomes. Nephrol Dial Transplant.

[9] Jhamb M., McNulty M.L., Ingalsbe G. (2016). Knowledge, barriers and facilitators of exercise in dialysis patients: a qualitative study of patients, staff and nephrologists. BMC Nephrol.

[10] Hargrove N., El Tobgy N., Zhou O. (2021). Effect of aerobic exercise on dialysis-related symptoms in individuals undergoing maintenance hemodialysis: a systematic review and meta-analysis of clinical trials. CJASN.

[11] Bernier-Jean A., Beruni N.A., Bondonno N.P. (2022). Exercise training for adults undergoing maintenance dialysis. Cochrane Kidney and Transplant Group. Cochrane Database Syst Rev.

[12] Slade S.C., Dionne C.E., Underwood M. (2016). Consensus on exercise reporting template (CERT): modified Delphi study. Physical Therapy.

[13] Akiba T., Matsui N., Shinohara S., Fujiwara H., Nomura T., Marumo F. (1995). Effects of recombinant human erythropoietin and exercise training on exercise capacity in hemodialysis patients. Artificial Organs.

[14] Graham-Brown M.P.M., March D.S., Young R. (2021). A randomized controlled trial to investigate the effects of intra-dialytic cycling on left ventricular mass. Kidney Int.

[15] Anastasia S., Evangelia K., Konstantinos F., Serafeim A., Asterios D. (2016). The effects of aquatic exercise on functional capacity and health-related quality of life in hemodialysis patients. J Clin Exp Nephrol.

[16] Groussard C., Rouchon-Isnard M., Coutard C. (2015). Beneficial effects of an intradialytic cycling training program in patients with end-stage kidney disease. Appl Physiol Nutr Metab.

[17] Yeh M.L., Wang M.H., Hsu C.C., Liu Y.M. (2020). Twelve-week intradialytic cycling exercise improves physical functional performance with gain in muscle strength and endurance: a randomized controlled trial. Clin Rehabil.

[18] Yabe H., Kono K., Ishikawa Y., Azekura H. (2020). Effects of intradialytic exercise for advanced aged patients undergoing hemodialysis: A randomized controlled trial. Nephrol Dial Transplant.

[19] Wu Y., He Q., Yin X., Cao S., Ying G. (2014). Effect of individualized exercise during maintenance haemodialysis on exercise capacity and health-related quality of life in patients with uraemia. J Int Med Res.

[20] Watanabe K., Kamijo Y., Yanagi M., Ishibashi Y., Harada T., Kohzuki M. (2021). Home-based exercise and bone mineral density in peritoneal dialysis patients: a randomized pilot study. BMC Nephrol.

[21] Vogiatzaki E., Michou V., Liakopoulos V. (2022). The effect of a 6-month intradialytic exercise program on hemodialysis adequacy and body composition: a randomized controlled trial. Int Urol Nephrol.

[22] van Vilsteren M.C., de Greef M.H., Huisman R.M. (2005). The effects of a low-to-moderate intensity pre-conditioning exercise programme linked with exercise counselling for sedentary haemodialysis patients in The Netherlands: results of a randomized clinical trial. Nephrol Dial Transplant.

[23] Uchiyama K., Washida N., Morimoto K. (2020). Effects of exercise on residual renal function in patients undergoing peritoneal dialysis: A post-hoc analysis of a randomized controlled trial. Ther Apher Dial.

[24] Tawney K.W., Tawney P.J., Hladik G. (2000). The life readiness program: a physical rehabilitation program for patients on hemodialysis. Am J Kidney Dis.

[25] Tabibi M.A., Salimian N. (2022). Pos-745 the effect of intradialytic concurrent exercise on metabolic acidosis, nutritional status, bone disorders and physical function in hemodialysis patients: a randomized controlled trial. Kidney International Reports.

[26] Tabibi M., Wilund K.R., Salimian N. (Preprint posted online March 21, 2023). The effect of intradialytic exercise on calcium, phosphorus and parathyroid hormone: a randomized controlled trial. medRxiv.

[27] Stringuetta Belik F., Oliveira E.S.V.R., Braga G.P. (2018). Influence of intradialytic aerobic training in cerebral blood flow and cognitive function in patients with chronic kidney disease: a pilot randomized controlled trial. Nephron.

[28] Salehi F., Mangolian Shahrbabaki P., Dehghan M., Amirihosseini M. (2022). Can passive pedaling improve sexual function in patients under hemodialysis? A randomized clinical trial. Ther Apher Dial.

[29] Rosa C., Nishimoto D.Y., Souza G.D.E. (2018). Effect of continuous progressive resistance training during hemodialysis on body composition, physical function and quality of life in end-stage renal disease patients: a randomized controlled trial. Clin Rehabil.

[30] Reboredo M.M., Neder J.A., Pinheiro B.V., Henrique D.M., Lovisi J.C., Paula R.B. (2015). Intra-dialytic training accelerates oxygen uptake kinetics in hemodialysis patients. Eur J Prev Cardiolog.

[31] Petraki M., Kouidi E., Grekas D., Deligiannis A. (2008). Effects of exercise training during hemodialysis on cardiac baroreflex sensitivity. Clinic Nephrol.

[32] Ouzouni S., Kouidi E., Sioulis A., Grekas D., Deligiannis A. (2009). Effects of intradialytic exercise training on health-related quality of life indices in haemodialysis patients. Clin Rehabil.

[33] Olvera-Soto M.G., Valdez-Ortiz R., Lopez Alvarenga J.C., Espinosa-Cuevas Md.e L. (2016). Effect of resistance exercises on the indicators of muscle reserves and handgrip strength in adult patients on hemodialysis. J Ren Nutr.

[34] Myers J., Chan K., Chen Y., Lit Y., Patti A., Massaband P. (2021). Effect of a Home-Based Exercise Program on Indices of Physical Function and Quality of Life in Elderly Maintenance Hemodialysis Patients. Kidney Blood Press Res.

[35] Moura S.R.G., Correa H.L., Neves R.V.P. (2020). Effects of resistance training on hepcidin levels and iron bioavailability in older individuals with end-stage renal disease: A randomized controlled trial. Exp Gerontol.

[36] Molsted S., Eidemak I., Sorensen H.T., Kristensen J.H. (2004). Five months of physical exercise in hemodialysis patients: Effects on aerobic capacity, physical function and self-rated health. Nephron - Clinical Practice.

[37] Michou V., Davioti M., Syrakou N., Liakopoulos V., Deligiannis A., Kouidi E. (2023 Jan 11). Effects of a Combined Intradialytic Exercise Training Program on Functional Capacity and Body Composition in Kidney Transplant Candidates. JFMK.

[38] Maynard L.G., de Menezes D.L., Liao N.S. (2019). Effects of exercise training combined with virtual reality in functionality and health-related quality of life of patients on hemodialysis. Games for Health Journal.

[39] Matsumoto Y., Furuta A., Furuta S. (2007). The impact of pre-dialytic endurance training on nutritional status and quality of life in stable hemodialysis patients (Sawada study). Renal Failure.

[40] Matsufuji S., Shoji T., Yano Y. (2015). Effect of chair stand exercise on activity of daily living: a randomized controlled trial in hemodialysis patients. J Ren Nutr.

[41] Martins do Valle F., Valle Pinheiro B., Almeida Barros A.A. (2020). Effects of intradialytic resistance training on physical activity in daily life, muscle strength, physical capacity and quality of life in hemodialysis patients: a randomized clinical trial. Disabil Rehabil.

[42] Marchesan M., Nunes V.G.D.S., Rombaldi A.J. (2014). Treinamento físico melhora a aptidão física e a qualidade de vida de pacientes em hemodiálise. Rev Bras Cineantropom Desempenho Hum.

[43] Manfredini F., Mallamaci F., D’Arrigo G. (2017). Exercise in patients on dialysis: a multicenter, randomized clinical trial. J Am Soc Nephrol.

[44] Krase A.A., Terzis G., Giannaki C.D. (2022). Seven months of aerobic intradialytic exercise training can prevent muscle loss in haemodialysis patients: an ultrasonography study. Int Urol Nephrol.

[45] Kouidi E.J., Grekas D.M., Deligiannis A.P. (2009). Effects of exercise training on noninvasive cardiac measures in patients undergoing long-term hemodialysis: a randomized controlled trial. Am J Kidney Dis.

[46] Kouidi E. (1997). Exercise renal rehabilitation program: Psychosocial effects. Nephron.

[47] Koufaki P., Mercer T.H., Naish P.F. (2002). Effects of exercise training on aerobic and functional capacity of end-stage renal disease patients. Clin Physiol Funct Imaging.

[48] Kim S., Park H.J., Yang D.H. (2022). An intradialytic aerobic exercise program ameliorates frailty and improves dialysis adequacy and quality of life among hemodialysis patients: a randomized controlled trial. Kidney Res Clin Pract.

[49] Huang M., Lv A., Wang J. (2020). The effect of intradialytic combined exercise on hemodialysis efficiency in end-stage renal disease patients: a randomized-controlled trial. Int Urol Nephrol.

[50] Huang H.Y., Hung K.S., Yeh M.L., Chou H.L., Yeh A.L., Liao T.Y. (2021). Breathing-based leg exercises during hemodialysis improve quality of life: A randomized controlled trial. Clin Rehabil.

[51] Lin C.H., Hsu Y.J., Hsu P.H. (2021). Effects of intradialytic exercise on dialytic parameters, health-related quality of life, and depression status in hemodialysis patients: a randomized controlled trial. Int J Environ Res Public Health.

[52] Greenwood S.A., Koufaki P., Macdonald J.H. (2021). Randomized Trial—PrEscription of intraDialytic exercise to improve quAlity of Life in Patients Receiving Hemodialysis. Kidney Int Rep.

[53] Goldberg A.P., Geltman E.M., Gavin J.R. (1986). Exercise training reduces coronary risk and effectively rehabilitates hemodialysis patients. Nephron.

[54] Frih B., Mkacher W., Bouzguenda A. (2017). Effects of listening to Holy Qur’an recitation and physical training on dialysis efficacy, functional capacity, and psychosocial outcomes in elderly patients undergoing haemodialysis. Libyan Journal of Medicine.

[55] Dong Z.J., Zhang H.L., Yin L.X. (2019). Effects of intradialytic resistance exercise on systemic inflammation in maintenance hemodialysis patients with sarcopenia: a randomized controlled trial. Int Urol Nephrol.

[56] Deus L.A., Corrêa H.D.L., Neves R.V.P. (2021). Are resistance training-induced BDNF in hemodialysis patients associated with depressive symptoms, quality of life, antioxidant capacity, and muscle strength? An insight for the muscle–brain–renal axis. Int J Environ Res Public Health.

[57] DePaul V., Moreland J., Eager T., Clase C.M. (2002). The effectiveness of aerobic and muscle strength training in patients receiving hemodialysis and EPO: A randomized controlled trial. Am J Kidney Dis.

[58] Corrêa H.L., Moura S.R.G., Neves R.V.P. (2020). Resistance training improves sleep quality, redox balance and inflammatory profile in maintenance hemodialysis patients: a randomized controlled trial. Sci Rep.

[59] Cooke A.B., Ta V., Iqbal S. (2018). The impact of intradialytic pedaling exercise on arterial stiffness: a pilot randomized controlled trial in a hemodialysis population. Am J Hypertens.

[60] Chen J.L., Godfrey S., Ng T.T. (2010). Effect of intra-dialytic, low-intensity strength training on functional capacity in adult haemodialysis patients: a randomized pilot trial. Nephrol Dial Transplant.

[61] Cheema B., Abas H., Smith B. (2007). Randomized controlled trial of intradialytic resistance training to target muscle wasting in ESRD: the Progressive Exercise for Anabolism in Kidney Disease (PEAK) study. Am J Kidney Dis.

[62] Bennett P.N., Hussein W.F., Matthews K. (2020). An exercise program for peritoneal dialysis patients in the united states: a feasibility study. Kidney Med.

[63] Abreu C.C., Cardozo L., Stockler-Pinto M.B. (2017). Does resistance exercise performed during dialysis modulate Nrf2 and NF-κB in patients with chronic kidney disease?. Life Sci.

[64] Abdelbasset W.K., Ibrahim A.A., Althomali O.W., Hussein H.M., Alrawaili S.M., Alsubaie S.F. (2022). Effect of twelve-week concurrent aerobic and resisted exercise training in non-dialysis day on functional capacity and quality of life in chronic kidney disease patients. Eur Rev Med Pharmacol Sci.

[65] Yurtkuran M., Alp A., Yurtkuran M., Dilek K. (2007). A modified yoga-based exercise program in hemodialysis patients: a randomized controlled study. Complementary Therapies in Medicine.

[66] Zhang F., Liao J., Zhang W. (2021). Effects of Baduanjin exercise on physical function and health-related quality of life in peritoneal dialysis patients: a randomized trial. Front Med.

[67] Cheng Y.J., Zhao X.J., Zeng W., Xu M.C., Ma Y.C., Wang M. (2020). Effect of intradialytic exercise on physical performance and cardiovascular risk factors in patients receiving maintenance hemodialysis: a pilot and feasibility study. Blood Purification.

[68] Harris L.K., Skou S.T., Juhl C.B., Jäger M., Bricca A. (2021). Recruitment and retention rates in randomised controlled trials of exercise therapy in people with multimorbidity: a systematic review and meta-analysis. Trials.

[69] Reynolds S.A., O’Connor L., McGee A. (2024). Recruitment rates and strategies in exercise trials in cancer survivorship: a systematic review. J Cancer Surviv.

[70] Cuschieri S. (2019). The CONSORT statement. Saudi J Anaesth.

[71] Courneya K.S., Segal R.J., Mackey J.R. (2007). Effects of aerobic and resistance exercise in breast cancer patients receiving adjuvant chemotherapy: a multicenter randomized controlled trial. J Clin Oncol.

[72] Linke S.E., Gallo L.C., Norman G.J. (2011). Attrition and adherence rates of sustained vs. intermittent exercise interventions. Ann Behav Med.

[73] Collado-Mateo D., Lavín-Pérez A.M., Peñacoba C. (2021). Key factors associated with adherence to physical exercise in patients with chronic diseases and older adults: an umbrella review. Int J Environ Res Public Health.

[74] Castillo G., Presseau J., Wilson M. (2022). Addressing feasibility challenges to delivering intradialytic exercise interventions: a theory-informed qualitative study. Nephrol Dial Transplantat.

