# Supplementary material for: Factors Influencing Recruitment, Retention, and Adherence Rates in Exercise Interventions in ESKD: A Scoping Review
Source: Kidney Med. 2024 Nov 14;7(1):100933. doi: 10.1016/j.xkme.2024.100933 (PMC11732533; doi:10.1016/j.xkme.2024.100933)
Supplement: Supplementary File (PDF) — Tables S1-S3. [file mmc1.pdf]

Table S1: Final Consensus on Exercise Reporting Template (CERT)

Item Category	Item Description
WHAT: Materials	Type of exercise equipment
WHO: Provider	Qualifications, teaching/supervising expertise, and/or training of the exercise instructor
HOW: Delivery	Whether exercises are performed individually or in a group
	Whether exercises are supervised or unsupervised
	Measurement and reporting of adherence to exercise
	Details of motivation strategies
	Decision rules for progressing the exercise program
	Each exercise is described so that it can be replicated (eg, illustrations, photographs)
	Content of any home program component
	Nonexercise components
	How adverse events that occur during exercise are documented and managed
WHERE: Location	Setting in which exercises are performed
WHEN, HOW MUCH: Dosage	Detailed description of the exercises (eg, sets, repetitions, duration, intensity)[a]
TAILORING: What, How	Whether exercises are generic ("one size fits all") or tailored to the individual[b]
	Decision rule that determines the starting level for exercise
HOW WELL: Planned, Actual	Whether the exercise intervention is delivered and performed as planned

a. The description of the exercises should enable the clinician, researcher, care-seeker or reviewer to know exactly how to administer or replicate the exercises that have been evaluated in a study.
b. Exercise programmes may be a predetermined and standardised set of exercises or tailored to the individual. A rationale, description and guide or system of decision rules for the tailoring, as well as implementation time points, should be provided.

Table S2: Table of Included Studies (Participant Characteristics)

		Experimental Group							***Control Group***						
tudy ID	*Country*	*# particip ants*	*Average age (Mean, STD or Median, IQR)*	*Sex (%M)*	*Duration of Dialysis (months)*	*% Hyperte nsion*	*% Diabetes*	*% Heart Disease*	*# of participants*	*Average age (Mean, STD or Median, IQR)*	*Sex (%M)*	*Duration of Dialysis (months)*	*% Hypertens ion*	*% Diabetes*	*% heart disease*
**kiba 1995 23)**	Japan	10	38.4, 9.5	20	73.8				10	40.6, 10.8	70	68.3			
**raham- rown 021(24)**	UK	65	55.5, 15.5	64.6	14.4	65.6	32.8	10.9	65	58.9, 14.9	81.5	15.6	67.7	43.1	13.8
**nastasia 016 (25)**	Greece	15	48, 11.3	86.6	Not specified				12	48.6, 15.4	91.7	Not specified			
**roussard 015 (26)**	France	10	66.5, 4.6	62.5	36.6				10	68.4, 3.7	80	41.2			
**hang 021(14)**	China	25	60.0, IQR 51.0 - 68.0	56	60				32	62.0, IQR 54.5 - 67.3	56.3	35.5			
**urtkuran 007(13)**	Turkey	20	38, 14.2	45	21.5				20	41, 9.97	35	20.2			
**eh 2020(27)**	Taipei	30	57.9, 13.2	63.3	63.5		57.1		32	53.9, 12.6	46.9	78.3		37.5	
**abe 021(28)**	Japan	44	78.7, 6.3	59.1	61.9		29.5		40	79, 6.7	52.5	70.9		20	
**Vu 2014(29)**	China	32	45, IQR 37-48	84.4	55.5				33	44, IQR 41-50	84.8	39.8			
**Vatanabe 021(30)**	Japan	26	66.2, 13.1	76.9	Not specified		30.8		27	64.00, 12.95	77.8	Not specified		33.3	
**ogiatzaki 022 (31)**	Greece	12	58.1, 14.3	66.7	98.4	25	41.7	33.3	12	57.4, 13.7	58.3	102	33.3	41.7	25
**anVilsteren 005 (32)**	Netherlands	53	52, 15	63.3	38.6				43	58, 16	69.8	46.8			
**Jchiyama 019(33)**	Japan	24	64.9, 9.2	79	43.2		25	29	23	63.2, 9.5	70	48		35	35
**awney 000(34)**	United States	51	Not specified	37.3	30		49		48	Not specified	43.8	31.5		50	
**abibi 2022 35)**	Iran	37	62, 13	57	29	27	43		37	65, 1	62	65	30	54	
**abibi 2023 36)**	Iran	22	61, 14	59	29	36	23		22	63, 1	68	25	41	14	
**tringuettaB lik 2018(37)**	Brazil	15	50.3, 17.2	46.6	26	86.6	20		15	57.8, 15.0	53.3	21.1	80	40	

**alehi 020(38)**	Iran	20	57.8, 9.2	65	43.5				17	54.6, 10.0	76.5	36.9			
**osa 018(39)**	Brazil	28	54.5, 11.9	57.1	18.5	6	9		24	57.1, 16.2	42.9	28.2	6	9	
**eboredo 015 (40)**	Brazil	12	50.7, 10.7	41.7	39.6	16.7			12	42.2, 13	41.7	57.6	8.3		
**etraki 008(41)**	Greece	22	50.1, 13.2	68.2	76.3				21	50.5, 14.4	81	72.8			
**uzouni 009(42)**	Greece	20	47.4, 15.7	73.7	92.4				15	50.5, 11.7	92.8	103.2			
**lvera-Soto 016(43)**	Mexico	30	28.5, IQR 23-46.5	47	12				31	29, IQR 19-38	61	18			
**yers 2021**	United States	15	66.3, 7.6	100	51	100	69	38.5	15	66.2, 6.7	100	48.6	100	66.7	33.3
**oura 020(44)**	Brazil	81	67.3, 3.2	56.8	Not specified	100	53		76	66.3, 3.9	52.6	Not specified	100	68.4	
**olsted 004(45)**	Denmark	22	59, IQR 25-58	63.6	23.5				11	48, IQR 23-58	72.7	17			
**ichou 023**(46)	Greece	15	53.3, 9.5	100	95.41	73.3	13.3	20	14	54.5, 9.9	100	96.7			
**aynard 019(46)**	Brazil	22	49, 15.2	60	62.7	65			23	43.9, 11.7	50	55.9	55	5	
**atsumoto 007(47)**	Japan	17	60.8, 9.5	29.4	148.8				32	57.2, 8.3	46.9	152.4			
**atsufuji 015 (48)**	Japan	12	69, IQR 61-78	67	168			58	15	69, IQR 64-79	73	180			
**artinsdoVa e 2020 (49)**	Brazil	12	49.3, 12.4	41.7	81.6				12	60.4, 10.6	66.7	46.8			
**archesan 014 (50)**	Brazil	11	45.1, 10.3	72.7	12				11	42.1, 11.1	72.7	12			
**anfredini 017 (51)**	Italy	104	63, 13	64	Not specified	77		15	123	64, 14	68	Not specified	70		17
**rase 2022 52)**	UK	21	66.0, 15.4	76.2	87.5				23	68.3, 11.1	43.5	64.7			
**ouidi 2009 53)**	United States	32	54.6, 8.9	60	75.6	73.3		30	31	53.2, 6.1	55.2	74.4	69		27.6
**ouidi 1997 54)**	Greece	20	49.6, 12.1	55	70.8				11	52.8, 10.2	57.1	74.4			
**oufaki 002 (55)**	UK	18	57.8, 14.3	72.2	41.4	40	20	45	15	51, 18.9	73.3	53.4	48	10	8
**im 022(56)**	Korea	18	57.6, 13.7	55.6	28.6				21	56.8, 12.3	47.6	68.9			

**Iuang 2020 57)**	China	24	43.8, 10.3	75	26				23	37.6, 10.3	68.8	43			
**Iuang 021(58)**	Taiwan	43	53.7, 10.0	72.5	Not specified	50	35		43	61.2, 10.2	65.1		35	48.8	
**Isu 2021(59)**	Taiwan	32	62, 9.5	68.8	80	87.5	43.8	12.5	32	62.1, 12.3	59.4	74	75	59.4	34.4
**reenwood 021(60)**	UK	135	60.5, 15.0	58.5	Not specified	74.8	38.5	10.4	145	59.8, 14.1	62.1	Not specified	80	40.7	11.7
**oldberg 986(61)**	United States	13	40, 14	61.5	23				12	36, 10	58.3	40			
**rih 017(62)**	Tunisia	21	64.2, 3.4	100	72.7				20	65.2, 3.1	100	73.6			
**ong 019(63)**	China	21	59.0, IQR 32.5 - 66.5	42.9	69	19	14.3		20	62.5, IQR 50.5 - 70.0	60	57.5	5	15	
**eus 021(64)**	Brazil	81	67.3, 3.2	47.4	54.1				76	66.3, 3.9	43.2	55.47			
**ePaul 002(65)**	Canada	20	55, 16	0.5	50.4		30	20	18	54, 14	76.5	55.2		35	35
**orr 020(66)**	Brazil	30	66.0, 4.0	100	60.7				25	65.7, 3.8	100	59.8			
**ooke 2018 57)**	Canada	10	58.2, 17.2	70	Not specified	100	30	20	10	52.5, 15.4	70	Not specified	100	40	20
**heng 020(15)**	China	67	54.6, 12.6	58.2	Not specified	11.9	20.9		65	55.8, 11.9	61.5	Not specified	4.6	29.2	
**hen 010(68)**	United States	22	71.1, 12.6	54.4	30	40.9	22.7		22	66.9, 13.4	50	56	27.3	45.5	
**heema 007(69)**	Australia	24	60, 15.3	70.8	Not specified	100	20.8	12.5	25	65, 12.9	68	Not specified	100	44	32
**ennett 020(70)**	United States	13	57.5, 16.3	61	Not specified	46	46		13	58.3, 16.7	46	Not specified	15	62	

**[illegible]breu [illegible]017(71)**	Brazil	25	45.7, 15.2	45.5	71.2	52	8		19	42.5, 13.5	38.5	70.1	32	8	
**[illegible]bdelbasset [illegible]022(72)**	Saudi Arabia	21	53.6, 17.8	33.3	28.6	52.8	42.8	4.7	22	48.7, 18.5	27.3	26.9	63.6	27.2	9.1

Table S3: Table of Included Studies (Intervention Characteristics)

Study ID	Recruitment Rate (%)	Retention Rate (%)	Adherence Rate (%)	How are exercises are performed?	Supervised or Unsupervised?	Setting	Frequency (times per week)	Session Duration (minutes)	Duration (weeks)	Exercise Type	Equipment Type
Akiba 1995 (13)		65		Individually			3	30 minutes or less	12	Aerobic	Cycle ergometer
Wu 2014(19)		94.2		Individually			3	30 minutes or less	12	Aerobic	Cycle ergometer
Graham-Brown 2021(14)	32	77.7	89.2	Individually	Supervised	Hospital	3	30 minutes or less	24	Aerobic	No equipment
Anastasia 2016 (15)		93.1		Group	Supervised		3	60 minutes or greater	16	Aerobic	Cycle ergometer
Groussard 2015 (16)		90		Individually	Supervised	Hospital	3	30 minutes or less	12	Aerobic	No equipment
Zhang 2021(64)	80.5	71.4	77.1	Individually	Non-Supervised	Home	5	30 minutes or less	12	Other: Bandujan	No equipment
Yurtkuran 2007(63)		95		Group	Supervised	Hospital	2	30 minutes or less	12	Other: Yoga	Cycle ergometer
Yeh 2020(17)	98.7	78.9		Individually	Supervised	Hospital	3	30 minutes or less	12	Aerobic	Cycle ergometer, TheraBand
Yabe 2021(18)	80.2	86.3		Individually	Supervised	Hospital	3	30 minutes or less	24	Combined Aerobic/Resistance	Cycle ergometer
Watanabe 2021(20)	92.3	72.2		Individually	Non-Supervised	Home	4	31-59 minutes	24	Combined Aerobic/Resistance	Cycle ergometer
Vogiatzaki 2022 (21)	85	70.6	93.2	Individually	Supervised	Hospital	3	60 minutes or greater	24	Aerobic	Cycle ergometer
vanVilsteren 2005 (22)	93.6	88.3		Individually	Supervised	Hospital	2	30 minutes or less	12	Combined Aerobic/Resistance	Theraband
Uchiyama 2019(23)	83.9	92	52	Individually	Non-Supervised	Home	2	30 minutes or less	12	Combined Aerobic/Resistance	No equipment
	45.6	82.8		Individually	Non-Supervised	Home	7	30 minutes or less	24	Other: Self-selected activities (household	No equipment

Tawney 2000(24)										chores, gardening, walking)	
Tabibi 2022 (25)	87.1	91.9		Group		Hospital	3	60 minutes or greater	24	Combined Aerobic/Resistance	Weight cuffs, dumbbells, elastic bands
Tabibi 2023 (26)	93.6	90.9	78.5	Group	Supervised	Hospital	3	31-59 minutes	24	Combined Aerobic/Resistance	Cycle ergometer
Stringuetta Belik 2018 (27)	42.2	88		Individually		Hospital	3	31-59 minutes	16	Aerobic	Electric exercise bike
Salehi 2020 (28)	56.3	68.5		Individually	Non-Supervised	Hospital	2	30 minutes or less	12	Aerobic	Unspecified resistance equipment
Rosa 2018 (29)	50.9	80	66.9	Group	Supervised	Hospital	3	31-59 minutes	12	Resistance	Semi-recumbent electromagnetically-braked cycle ergometer
Reboredo 2015 (30)	70	85.7		Individually	Supervised	Hospital	3	60 minutes or greater	12	Aerobic	Cycle ergometer, therabands
Petraki 2008 (31)		84.6		Group	Supervised	Hospital	3	60 minutes or greater	28	Combined Aerobic/Resistance	Cycle, weights, therabands
Ouzouni 2009 (32)		94.3		Individually	Supervised	Hospital	3	60 minutes or greater	40	Combined Aerobic/Resistance	Resistance bands, ankle weight belts
Olvera-Soto 2016 (33)	79.2	96.7		Group	Supervised	Hospital	2	31-59 minutes	12	Resistance	Hand-held weights, Thera-bands, portable cycle ergometers
Myers 2021 (34)	20	90.3		Individually	Non-Supervised	Hospital, Home		31-59 minutes	12	Combined Aerobic/Resistance	Dumbells
Moura 2020(35)	85.6	77.7		Group	Supervised	Hospital	3	60 minutes or greater	24	Resistance	No equipment
Molsted 2004(36)	33	39.4		Individually	Supervised		2	60 minutes or greater	20	Aerobic	Stationary bicycles, resistance TheraBands, dumbbells, stress balls
Michou 2023(37)	95.2	75		Individually	Supervised	Hospital	3	60 minutes or greater	16	Combined Aerobic/Resistance	Cycle ergometer, thera-band, ankle weight, Nintendo Wii
Maynard 2019(38)	86.5	91			Supervised	Hospital	3	31-59 minutes	12	Combined Aerobic/Resistance	Stationary cycle
Matsumoto 2007(39)		77.3		Individually	Supervised	Hospital	3	60 minutes or greater	52	Aerobic	Chair
Matsufuji 2015 (40)	48.9	73.9			Supervised	Other: Rehab room	3	60 minutes or greater	12	Resistance	Weighted ankle cuffs, dumbells
MartinsdoValle 2020 (41))	55.8	41.7	85.8	Group	Supervised		3		12	Resistance	Cycle ergometer
Marchesan 2014 (42)	84.8	100		Individually		Hospital	3	31-59 minutes	17	Aerobic	Seiko DM50 metronome
Manfredini 2017 (43)	59.56	68.87	83	Individually	Non-Supervised	Home	6	30 minutes or less	24	Aerobic	Bedside cycle ergometer
Krase 2022 (44)	98	87.5		Individually	Supervised	Hospital	3	60 minutes or greater	28	Aerobic	Bicycle, Theraband

Kouidi 2009 (45)	40	93.7	88.3	Individually	Supervised	Hospital	3	60 minutes or greater	40	Combined Aerobic/Resistance	Cycle ergometer
Kouidi 1997 (46)		83.3	78	Individually	Supervised	Other: Sports medicine lab	3	60 minutes or greater	24	Combined Aerobic/Resistance	Cycle ergometer
Koufaki 2002 (47)		68.8		Individually	Supervised	Gym	3	31-59 minutes	12	Aerobic	Cycle ergometer
Kim 2022(48)	75	85.7		Individually	Supervised	Hospital	3	31-59 minutes	12	Aerobic	Cycle ergometer
Huang 2020 (49)	75.8	68		Individually	Supervised	Hospital	3	60 minutes or greater	24	Combined Aerobic/Resistance	No equipment
Huang 2021(50)	76.8	96.5		Group	Supervised	Hospital	3	60 minutes or greater	12	Combined Aerobic/Resistance	Lower-limb ergometer (WP-698, Magnetic Mini Bike, Taiwan)
Lin 2021(51)	39.3	89.1		Individually	Supervised	Hospital	3	60 minutes or greater	12	Aerobic	Cycle ergometer
Greenwood 2021(52)	36.8	66.3	18	Individually	Supervised	Hospital	3	31-59 minutes	24	Aerobic	Cycle ergometer
Goldberg 1986(53)		92		Individually	Supervised		3	31-59 minutes	36	Aerobic	Multigym, cycle ergometer, treadmill, heart rate monitor
Frih 2017(54)	92.6	75		Individually	Supervised		4	60 minutes or greater	16	Combined Aerobic/Resistance	Ankle weight, quadriceps training board, elastic ball
Dong 2019(55)		91.3		Individually		Hospital	3	60 minutes or greater	12	Resistance	e-Lastic, weighted cuffs, dumbbells
Deus 2021(56)	85.6	80.2		Individually	Supervised	Hospital	3	60 minutes or greater	24	Resistance	Response Seated Leg Curl Thigh Extension pulley weight system,
DePaul 2002(57)	35.2	76.3		Individually	Supervised	Hospital	3	31-59 minutes	12	Combined Aerobic/Resistance	Thera-band, dumbbells, fixed weights
Correa 2020(58)	88.6	62.5		Individually	Supervised	Hospital	3	31-59 minutes	12	Resistance	No equipment
Cooke 2018 (59)	67.5	66.7	60	Individually	Non-Supervised	Hospital	3	31-59 minutes	16	Aerobic	Free weights
Cheng 2020(67)	78.1			Group		Hospital	3	30 minutes or less	104	Resistance	Free weights
Chen 2010(60)	20	88	89		Supervised	Hospital	2		24	Resistance	Free-weight dumbells, weighted ankle cuffs , Thera-band tubing
Cheema 2007(61)	63.6	83.3		Individually	Supervised	Hospital	3		12	Resistance	Chair, Pinch-grip dynamometer
Bennett 2020(62)	63	72	77	Individually	Non-Supervised	Home	3	60 minutes or greater	12	Combined Aerobic/Resistance	Ankle cuffs, elastic bands (Theraband)

Abreu 2017(63)		72	90.6	Individually	Supervised	Hospital	3	60 minutes or greater	12	Resistance	Treadmill, cycle ergometer, Polar Electro heart rate monitor, weights
Abdelbasset 2022(64)	67.6	84		Individually		Other: Physiotherapy clinic	3	31-59 minutes	12	Combined Aerobic/Resistance	
